# Clinical, Genetic, and Histological Characterization of Patients with Rare Neuromuscular and Mitochondrial Diseases Presenting with Different Cardiomyopathy Phenotypes

**DOI:** 10.3390/ijms24109108

**Published:** 2023-05-22

**Authors:** Emanuele Monda, Michele Lioncino, Martina Caiazza, Vincenzo Simonelli, Claudia Nesti, Marta Rubino, Alessia Perna, Alfredo Mauriello, Alberta Budillon, Vincenzo Pota, Giorgia Bruno, Antonio Varone, Vincenzo Nigro, Filippo Maria Santorelli, Giuseppe Pacileo, Maria Giovanna Russo, Giulia Frisso, Simone Sampaolo, Giuseppe Limongelli

**Affiliations:** 1 Inherited and Rare Cardiovascular Disease Unit, Department of Translational Medical Sciences, University of Campania Luigi Vanvitelli, AORN dei Colli, Monaldi Hospital, 81031 Naples, Italy; emanuelemonda@me.com (E.M.); michelelioncino@icloud.com (M.L.); martina.caiazza@yahoo.it (M.C.); rubinomarta@libero.it (M.R.); alessiaperna@hotmail.it (A.P.); alfredo.mauriello93@libero.it (A.M.); 2 Neurology Unit, AORN dei Colli, Monaldi Hospital, 81031 Naples, Italy; vincenzo.simonelli@ospedalideicolli.it; 3 Molecular Medicine, IRCCS Stella Maris Foundation, 56128 Pisa, Italy; claudia.nesti@fsm.unipi.it (C.N.); filippo.santorelli@fsm.unipi.it (F.M.S.); 4 Department of Advanced Medical and Surgical Sciences, 2nd Division of Neurology, Center for Rare Diseases and InterUniversity Center for Research in Neurosciences, University of Campania Luigi Vanvitelli, Via Sergio Pansini, 5, 80131 Naples, Italy; albybudillon@hotmail.com (A.B.); simone.sampaolo@unicampania.it (S.S.); 5 NeuroMuscular Omnicentre (NEMO), AORN dei Colli, Monaldi Hospital, 80131 Naples, Italy; vincenzo.pota@ospedalideicolli.it; 6 Pediatric Neurology Unit, Department of Neurosciences, Santobono-Pausilipon Children’s Hospital, 80122 Naples, Italy; giorgiabruno990@gmail.com (G.B.); a.varone@santobonopausilipon.it (A.V.); 7 Department of Precision Medicine, University of Campania Luigi Vanvitelli, Via Luigi De Crecchio 7, 80138 Naples, Italy; vincenzo.nigro@unicampania.it; 8 Telethon Institute of Genetics and Medicine, Via Campi Flegrei 34, 80078 Pozzuoli, Italy; 9 Heart Failure Unit, Department of Cardiology, AORN dei Colli, Monaldi Hospital, 80131 Naples, Italy; gpacileo58@gmail.com; 10 Paediatric Cardiology Unit, University of Campania Luigi Vanvitelli, AORN dei Colli, Monaldi Hospital, 81100 Caserta, Italy; mgiovannarusso@gmail.com; 11 Department of Molecular Medicine and Medical Biotechnology, University of Naples “Federico II”, 80138 Naples, Italy; gfrisso@unina.it; 12 Institute of Cardiovascular Sciences, University College of London and St. Bartholomew’s Hospital, Gower St, London WC1E 6DD, UK

**Keywords:** cardiomyopathy, neuromuscular disease, genetic testing

## Abstract

Cardiomyopathies are mostly determined by genetic mutations affecting either cardiac muscle cell structure or function. Nevertheless, cardiomyopathies may also be part of complex clinical phenotypes in the spectrum of neuromuscular (NMD) or mitochondrial diseases (MD). The aim of this study is to describe the clinical, molecular, and histological characteristics of a consecutive cohort of patients with cardiomyopathy associated with NMDs or MDs referred to a tertiary cardiomyopathy clinic. Consecutive patients with a definitive diagnosis of NMDs and MDs presenting with a cardiomyopathy phenotype were described. Seven patients were identified: two patients with ACAD9 deficiency (*Patient 1* carried the c.1240C>T (p.Arg414Cys) homozygous variant in *ACAD9*; *Patient 2* carried the c.1240C>T (p.Arg414Cys) and the c.1646G>A (p.Ar549Gln) variants in *ACAD9*); two patients with *MYH7*-related myopathy (*Patient 3* carried the c.1325G>A (p.Arg442His) variant in *MYH7*; *Patient 4* carried the c.1357C>T (p.Arg453Cys) variant in *MYH7*); one patient with desminopathy (*Patient 5* carried the c.46C>T (p.Arg16Cys) variant in *DES*); two patients with mitochondrial myopathy (*Patient 6* carried the m.3243A>G variant in *MT-TL1*; *Patient 7* carried the c.253G>A (p.Gly85Arg) and the c.1055C>T (p.Thr352Met) variants in *MTO1*). All patients underwent a comprehensive cardiovascular and neuromuscular evaluation, including muscle biopsy and genetic testing. This study described the clinical phenotype of rare NMDs and MDs presenting as cardiomyopathies. A multidisciplinary evaluation, combined with genetic testing, plays a main role in the diagnosis of these rare diseases, and provides information about clinical expectations, and guides management.

## 1. Introduction

Cardiomyopathies are a heterogeneous group of diseases affecting the heart muscle that are associated with structural and functional myocardial abnormalities that cannot be solely explained by coronary artery disease, valvular heart disease, congenital heart disease, or hypertension [1,2].

Although cardiomyopathies can be caused by inherited or acquired mechanisms, they are mostly determined by genetic mutations affecting either cardiac muscle cell structure (e.g., sarcomere proteins, cytoskeleton, transmembrane proteins, and desmosomal) or function (e.g., calcium receptors, ion channels, and cellular bioenergetics) [3,4,5,6,7,8]. Moreover, several environmental factors (e.g., alcohol or drugs exposure, pregnancy, diabetes) may trigger a cardiomyopathy phenotype [9,10,11]. Nevertheless, cardiomyopathies may also be part of complex clinical phenotypes in the spectrum of neuromuscular (NMDs) or mitochondrial diseases (MDs) [2,12,13].

Differentiating isolated cardiomyopathies from genocopies/phenocopies is relevant since the latter may require tailored cardiac and non-cardiac management [14,15,16,17]. Moreover, patients with NMDs and MDs often have worse outcomes compared with sarcomeric/idiopathic cardiomyopathies [15,18].

Due to the diverse etiology of these conditions, the morphological diagnosis of a cardiomyopathy should prompt a systematic search for its underlying cause. In many patients, this work-up should include specialized laboratory testing and, often, genetic investigations [12,19]. Electromyography (EMG) and muscle biopsy (MB) may represent essential tools for the diagnosis of NMDs, showing high diagnostic accuracy in patients with a suspected myopathy [20]. However, genetic testing remains crucial for the diagnostic work-up, allowing for a definitive diagnosis and guiding specific therapies [21].

We recently reported the prevalence of NMDs associated with cardiomyopathy using a combined clinical, genetic, and neuromuscular screening in our study population [22]. In this study, we sought to describe the clinical, molecular, and histological characteristics of those consecutive cardiomyopathy patients.

## 2. Results

Seven patients with a definitive diagnosis of NMDs or MDs were identified: two patients with ACAD9 deficiency (*Patient 1* and *Patient 2*); two patients with *MYH7*-related myopathy (*Patient 3* and *Patient 4*); one patient with desminopathy (*Patient 5*); two patients with mitochondrial myopathy (one with *MT-TL1* mutation (*Patient 6*) and one with *MTO1* mutation (*Patient 7*)).

### 2.1. ACAD9

Two patients harbored variants in *ACAD9* and presented a hypertrophic cardiomyopathy (HCM) phenotype. 

#### 2.1.1. Patient 1

**Figure 1:**
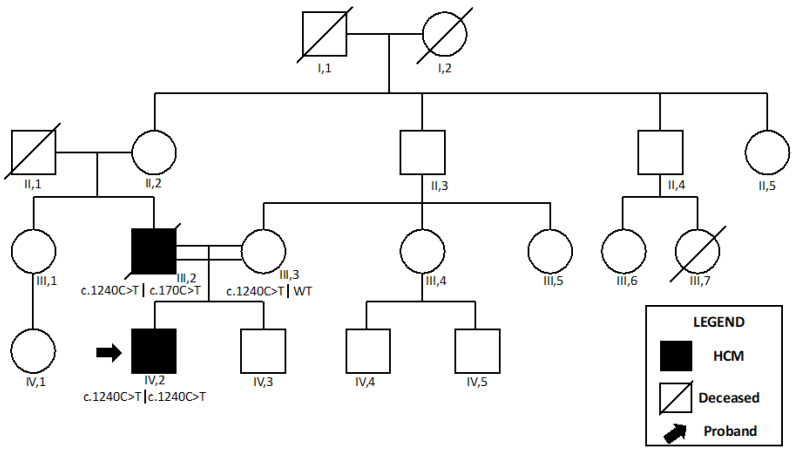
Pedigree of the *Patient 1*. *Patient 1* is shown as IV,2. A family member (III,7) died suddenly at 36 years old. However, his father (II,4) refused to perform a comprehensive cardiovascular evaluation. Abbreviations: HCM, hypertrophic cardiomyopathy.

*Patient 1* was the son of consanguineous parents (Figure 1).

At birth, he experienced severe respiratory distress requiring intensive care therapy. From the age of 18 months, because of exercise intolerance and easy fatigability, he was diagnosed with severe, non-obstructive HCM with marked asymmetric hypertrophy of the basal septum, the apex, and the papillary muscles. 

During his second year of life, his father died suddenly. At physical examination, the proband showed severe exercise intolerance, bilateral scapulothoracic winging, and hypotrophy of the scapular cingulum and of the gastrocnemius muscles. Cardiac magnetic resonance (CMR) showed normal systolic function and the absence of late gadolinium enhancement (LGE).

**Figure 2:**
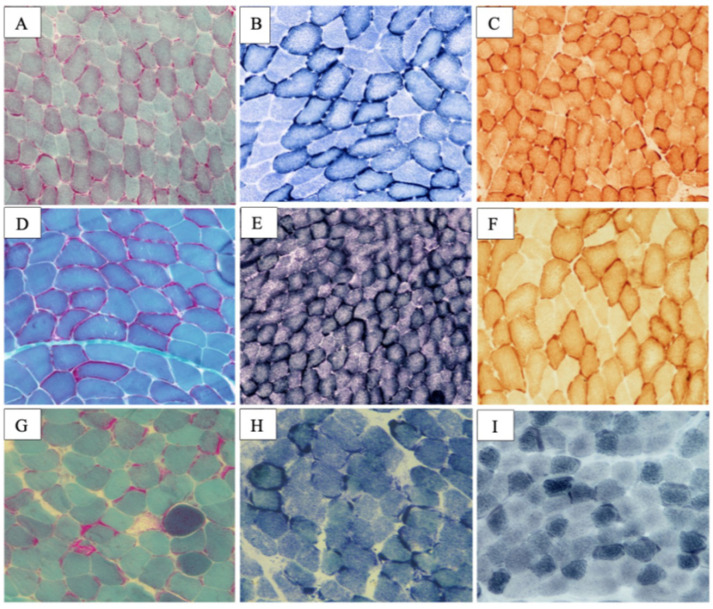
Histopathologic findings in ACAD9 deficiency. *Patient 1* muscle biopsy showed mild caliber heterogeneity and multiple subsarcolemmal and interfibrillar mitochondrial aggregates: Gomori trichomic stain (**A**); succinyl-dehydrogenase histochemical stain (SDH) (**B**); cyclooxygenase histochemical (COX) stain (**C**). Father of the *Patient 1* muscle biopsy specimens: Gomori trichromic stain (**D**); SDH (**E**); COX (**F**). Marked lipid accumulation at Sudan stain was evident at subsarcolemmal level (**E**), suggesting an oxidative disorder. *Patient 2* muscle biopsy: Trichomic Gomori stain evidenced red-ragged fibers (**G**); SDH stain showed increased mitochondria accumulation (**H**); Sudan black stain showed marked intracellular lipid accumulation, both at subsarcolemmal and intermyofibrillar levels (**I**). Size scale bars: (**A**–**D**,**F**–**I**) = magnification ×20; (**E**) = magnification ×10.

The MB showed mild fiber heterogeneity and subsarcolemmal and interfibrillar aggregates of mitochondria (Figure 2A–C). Marked lipid accumulation was detected at the intermyofibrillar and subsarcolemmal levels. Some fibers showed significantly impaired cytochrome oxidase activity, which was more evident in central core regions. Histology data were supported by the significant reduction in complex I activity at spectrophotometry. 

Supplementation with carnitine and riboflavin was started based on these findings. 

Because of multiple episodes of syncope, an internal loop recorder (ILR) was implanted. However, it failed to detect any arrhythmic event during follow-up.

The genetic testing demonstrated a homozygous missense variant in the *ACAD9* gene (c.1240C>T, p.Arg414Cys), which was classified as pathogenic according to American College of American Genetics and Genomics (ACMG) criteria (Table 1). His mother was found to be a heterozygous carrier of the same variant, and her physical examination was unremarkable.

His father was found to have severe, non-obstructive HCM with increased creatine kinase and signs of myopathy during cascade screening. His 12-lead ECG showed a short PR interval and frequent polymorphic premature ventricular beats. Furthermore, a short run of non-sustained ventricular tachycardia was detected at 24 h Holter monitoring. 

Although clinical examination did not show muscle weakness, subsequent EMG demonstrated polyphasic motor potentials of short duration and low amplitude. MB identified signs of mitochondrial proliferation in some cells associated with multiple subsarcolemmal aggregates, which resulted positive at the Gomori trichromic stains, and the succinate dehydrogenase (SDH), cyclooxygenase (COX), and histochemical stains (Figure 2D–F). Deficiency of mitochondrial complex I was confirmed on his muscle specimen.

Molecular analysis initially demonstrated the heterozygous missense variant in *ACAD9* (c.1240C>T, p.Arg414Cys) which had been previously identified in his son. 

Etiological work-up and genetic testing were performed after the patient married a consanguineous woman and gave birth to a child. Because of an unwitnessed out-of-hospital cardiac arrest, the patient died suddenly at 42 years old. Characterization of a copy DNA (cDNA) library was obtained by muscle tissue post-mortem. Interestingly, a second mutation in *ACAD9* (c.170C>T, p.Pro57Leu) was identified, which was classified as a variant of uncertain significance (VUS) according to ACMG criteria.

#### 2.1.2. Patient 2

*Patient 2* was diagnosed with non-obstructive HCM at the age of 12. He showed a short PR interval at baseline ECG. Although EMG was normal, impaired muscle strength with bilateral proximal weakness was present at clinical evaluation. The MB detected subsarcolemmal aggregates of mitochondria. Red-ragged fibers were identified at the Trichomic Gomori stains (Figure 2G). Marked intracellular lipid accumulation, both at subsarcolemmal and intermyofibrillar level, was evident at the Sudan stains (Figure 2I). 

**Figure 3:**
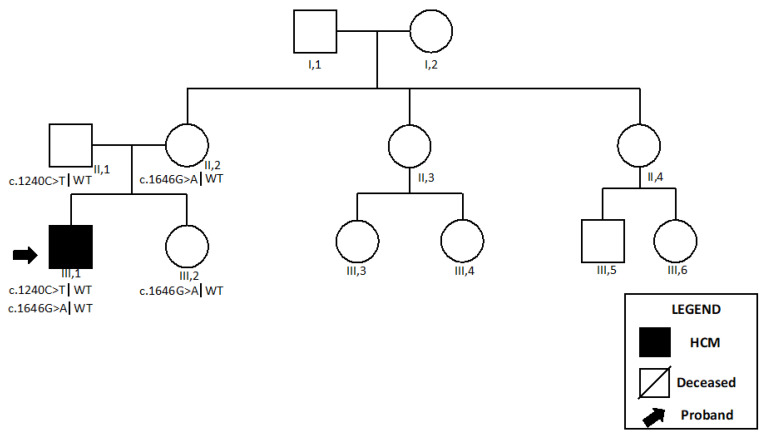
Pedigree of the *Patient 2*. *Patient 2* is shown as III,1. Abbreviations: HCM, hypertrophic cardiomyopathy.

The genetic testing showed a compound heterozygosis. He carried a missense variant in *ACAD9* (c.1240C>T, p.Arg414Cys), previously described, and a second variant (c.1646G>A, p.Ar549Gln) classified as VUS (Table 1). Co-segregation studies documented that the patient had inherited the c.1240C>T variant from his father and the c.1646G>A was present in the proband’s mother and sister. No first-degree family member showed any cardiovascular abnormality (Figure 3).

### 2.2. MYH7-Related Myopathy

#### 2.2.1. Patient 3

**Figure 4:**
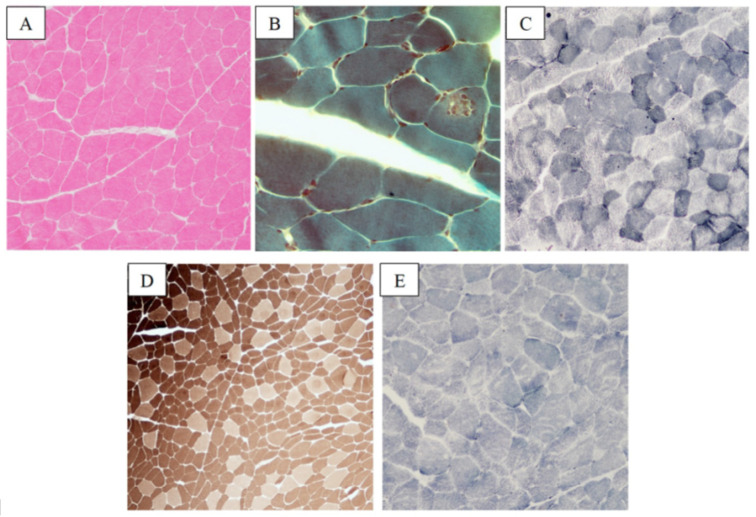
Muscle biopsy of patients with *MYH7* myopathy. *Patient 3*: marked heterogeneity among muscle fibers, with evidence of myofibrillar necrosis (**A**). H-E stain showing the absence of typical eosinophilic inclusions described in *MYH7* typical myopathy (**B**). Trichromic Gomori stain (**C**,**E**) SDH stains showing diffuse mini-cores. *Patient 4:* ATPase stains showing prevalence of small type 2 fibers (**D**). Size scale bars: (**A**,**C**,**E**) = magnification ×20; (**B**) = magnification ×40; (**D**) = magnification ×10.

*Patient 3* had a family history of cardiovascular disease and was diagnosed at 27 years old with dilated cardiomyopathy (DCM). Her neurological evaluation demonstrated a waddling gait, hypotrophy of deltoid muscles, and palpebral ptosis due to orbicularis oculi muscle hypotonia. Her EMG was positive for a myopathic pattern. MB showed marked heterogeneity among muscle fibers, with evidence of myofibrillar necrosis and endomysial perivascular leukocytes and diffuse mini cores. Eosinophilic inclusions described in muscle biopsies of typical *MYH7* patients were absent [23] (Figure 4A–C).

**Figure 5:**
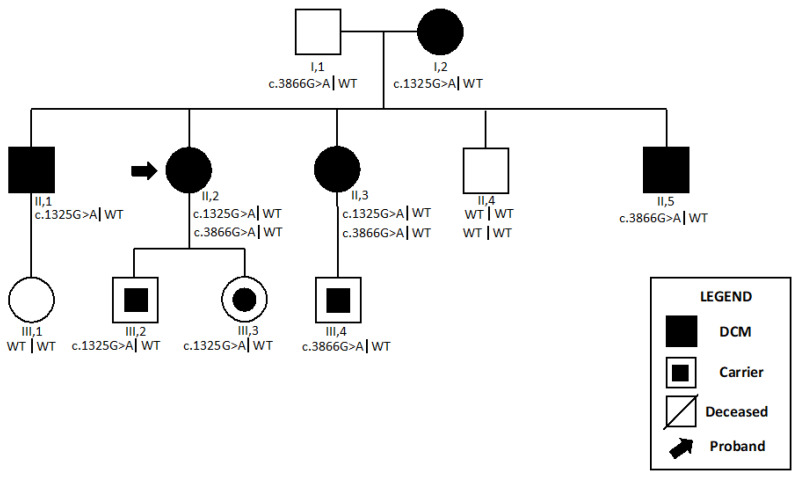
Pedigree of *Patient 3*. *Patient 3* is shown as II,2. The NGS sequencing demonstrated a pathogenic variant (*MYH7*, c.1325G>A) and a VUS (*MYH7*, c.3866G>A). The sister of the proband (II,3) showed similar cardiac phenotype and carried both variants. Patient’s brother (II,5) was affected by DCM and his genotype was found to be wild type for *MYH7* c.1325G>A variant, whereas he carried *MYH7* c.3866G>A variant. Abbreviations: DCM, dilated cardiomyopathy.

Genetic testing sequencing demonstrated a heterozygous missense variant in *MYH7* (c.1325G>A, p.Arg442His), which was classified as pathogenetic according to ACMG criteria, and a second heterozygous VUS (c.3866G>A, p.Arg1289Gln) (Table 1). The sister of the proband (II,3, Figure 5) showed a similar cardiac phenotype and carried both mutations. Interestingly, the patient’s brother (II,7) was affected by DCM and carried the c.3866G>A variant. 

#### 2.2.2. Patient 4

*Patient 4* came to our attention for paroxysmal, high-grade atrioventricular block and family history of HCM. Mild muscle weakness and waddling gait were present at physical examination. Her echocardiogram showed severely reduced systolic function (left ventricular ejection fraction = 30%), prominent apical trabeculations fulfilling diagnostic criteria for left ventricular non-compaction (LVNC), and severe mitral regurgitation. 

According to current guidelines [24], she underwent implantable cardioverter-defibrillator (ICD) implantation for primary prevention of sudden cardiac death (SCD). The cardiopulmonary exercise test demonstrated a severe impairment in functional capacity (vO_2_ = 7 mL/Kg min). The MB demonstrated increased variability in muscle myofibril distribution, the predominance of small type 2 fibers and diffuse mini cores. 

**Figure 6:**
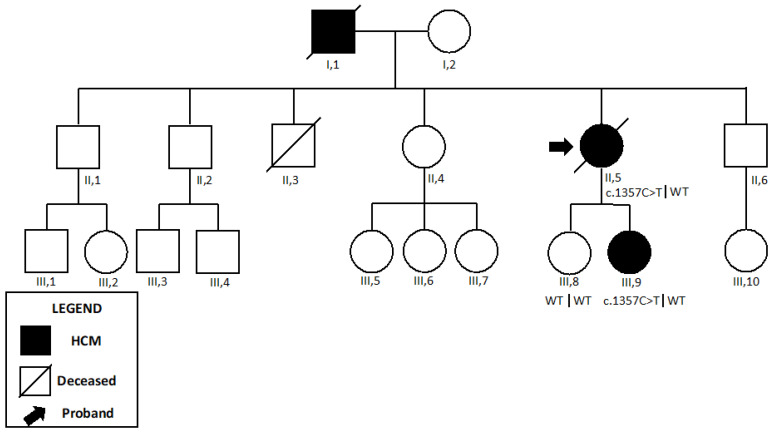
Pedigree of the *Patient 4*. *Patient 4* is shown as II,5. NGS sequencing revealed the pathogenic variant c.1357C>T in *MYH7* in the proband (II,5) and in her daughter (III,9). Abbreviations: HCM, hypertrophic cardiomyopathy.

Genetic testing revealed a heterozygous missense variant in *MYH7* (c.1357C>T, p.Arg453Cys), which was classified as pathogenic, according to ACMG criteria (Table 1). Because the patient experienced multiple hospitalizations for heart failure, she was listed for heart transplantation, after careful evaluation of the Heart Team. The family evaluation showed that the patient’s daughter (III,9) was affected with HCM and carried the same mutation. Physical examination and genetic testing were negative in the other first-degree relatives (Figure 6).

### 2.3. Desminopathy

*Patient 5* was referred by the general practitioner for the occurrence of proximal muscle weakness associated with abnormal gait and deltoid hypotonia. At 40 years old, she was diagnosed with DCM and heart failure therapy was started. Her family history was unremarkable. Her 12-lead ECG showed a first-degree atrioventricular block and sinus pauses without any history of syncope. Echocardiography showed a dilated left ventricle with severely reduced ejection fraction and restrictive filling physiology with increased pulmonary pressures.

**Figure 7:**
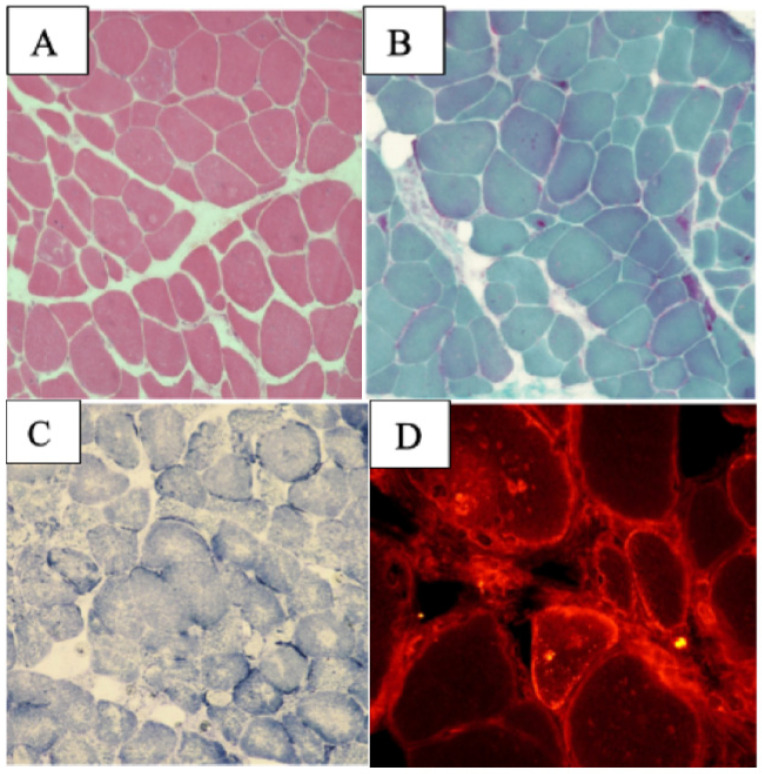
Histopathological findings in myofibrillar myopathy caused by desmin intracellular accumulation. H-E stain showing increased endomysial connective tissue deposition and rare moth-eaten fibers (**A**). Dark-blue, basophil inclusions were detected at Gomori stain (**B**). Cores were identified at SDH histochemical stain (**C**). Immunoreaction stain with antibodies for desmin was positive (**D**). Size scale bars: (**A**–**C**) = magnification ×20; (**D**) = magnification ×40.

The MB showed a trend towards type grouping with hypertrophy of type 1 fibers and hypotrophy of type 2 fibers, diffuse moth-eaten fibers and increased endomysial connective tissue deposition. Dark-blue basophil inclusions were identified at the Gomori stain. The SDH histochemical stain showed central core fibers. Immunoreactive staining with antibodies for desmin was positive (Figure 7). 

The genetic testing demonstrated a heterozygous missense variant in *DES* (c.46C>T, p.Arg16Cys) [25] which was classified as likely pathogenic according to ACMG criteria (Table 1). Unfortunately, genetic cascade screening was not performed. Because of worsening heart failure symptoms requiring hospital admissions, the patient underwent heart transplantation. 

### 2.4. Mitochondrial Encephalopathy, Lactic Acidosis, and Stroke-Like Episodes (MELAS) Syndrome

*Patient 6* had a family history of HCM (his mother and three siblings). At 30 years old, he had been diagnosed with bilateral deafness and, because of end-stage chronic kidney disease, he had started hemodialysis at 45 years old. Three years later, he was listed for a kidney transplant and, during this hospital stay, non-obstructive, hypokinetic HCM with reduced ejection fraction and biventricular involvement was diagnosed. Through 24 h Holter monitoring, multiple episodes of atrioventricular re-entrant tachycardia were revealed, and an electrophysiological study was indicated. Radiofrequency ablation of the left lateral accessory pathway was performed, and antiarrhythmic therapy was withdrawn in the absence of further episodes of tachycardia. 

After three months, 24 h Holter monitoring identified multiple runs of non-sustained ventricular tachycardia, and, after a multidisciplinary discussion, the decision to implant an ICD was pursued. After ICD implantation, the patient was referred to our Unit. 

**Figure 8:**
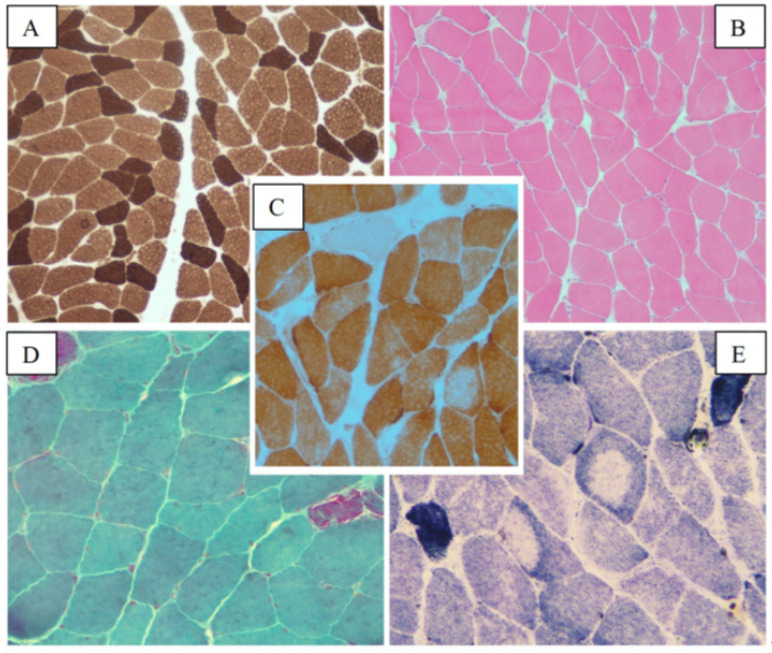
Histopathologic findings in a patient with MELAS syndrome. ATPase histochemical reaction showing marked fiber heterogeneity with atrophy of type 2 fibers (**A**). Red-ragged fibers were evident at Gomori trichomic stains (**D**). COX and SDH histochemical stains showing marked reduction or absence of enzymatic activity. In central cores (**C**,**E**). H-E stain (**B**). Size scale bars: (**A**–**E**) = magnification ×20.

Clinical evaluation showed a severe reduction in muscle strength and a myopathic pattern at EMG. MB was performed showing marked heterogeneity of muscle cells, with small groups of hypo-atrophic fibers, mainly represented by type 2 fibers, and subsarcolemmal mitochondrial aggregates which resulted positive at immunohistochemical stains for SDH and COX. Red-ragged fibers were evident at the Gomori stain. The immunohistochemical stains for SDH and COX showed diffuse central cores with lack of enzymatic activity (Figure 8).

Subsequently, genetic testing was indicated, and family screening was proposed to identify potentially at-risk relatives. The genetic testing identified a missense variant in *MT-TL1 (*m.3243A>G) and the patient was diagnosed as having mitochondrial encephalomyopathy, lactic acidosis, and stroke-like episodes (MELAS) (Table 1). In this case, the degree of heteroplasmia was not assessed. Although her past medical history was significant for multiple stroke episodes, the patient did not agree to undergo brain magnetic resonance imaging because of claustrophobia. 

### 2.5. Mitochondrial tRna Translation Optimization (MTO1)

*Patient 7* was diagnosed at 11 years old with non-obstructive HCM. His family history was unremarkable. At 18 years old, he experienced a syncopal episode during exertion, so he was admitted to the emergency department. Exercise echocardiography excluded the presence of provocable left ventricular outflow tract obstruction. A head-up tilt test and electrophysiological study were performed to assess the syncope etiology. Although the electrophysiological study failed to demonstrate any inducible arrhythmia, the head-up tilt test documented a tilt-induced, non-asystolic vasodepressive syncope. Therefore, the patient was reassured about the benign course of this condition and behavioral maneuvers were indicated.

**Figure 9:**
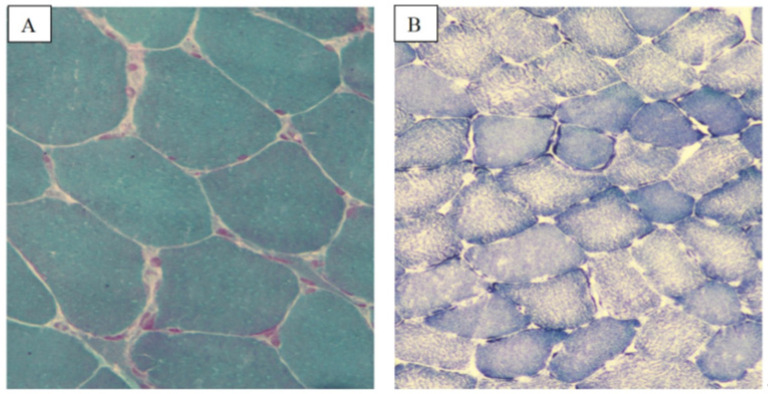
Histopathologic findings in a patient with *MTO1*-associated HCM. Gomori trichromic stains showing rare atrophic fibers (**A**). SDH histochemical stain showing subsarcolemmal aggregates and diffuse reduction in enzymatic activity in the central core (**B**). Size scale bars: (**A**) = magnification ×40; (**B**) = magnification ×20.

Because of recurrent fainting episodes, the patient underwent brain magnetic resonance imaging, which showed a small area with low signal intensity in T1-weighted and hyperintense in T2 sequences which seemed compatible with a small gliotic focus on the hypothalamic region. Because of reduced muscle strength and abnormal EMG, the MB was indicated. Pathologic findings suggested mild mitochondrial myopathy. In particular, rare eosinophilic atrophic fibers were found at the Gomori trichomic, and the SDH histochemical stain showed diffuse mini-cores and subsarcolemmal aggregates of mitochondria (Figure 9).

**Figure 10:**
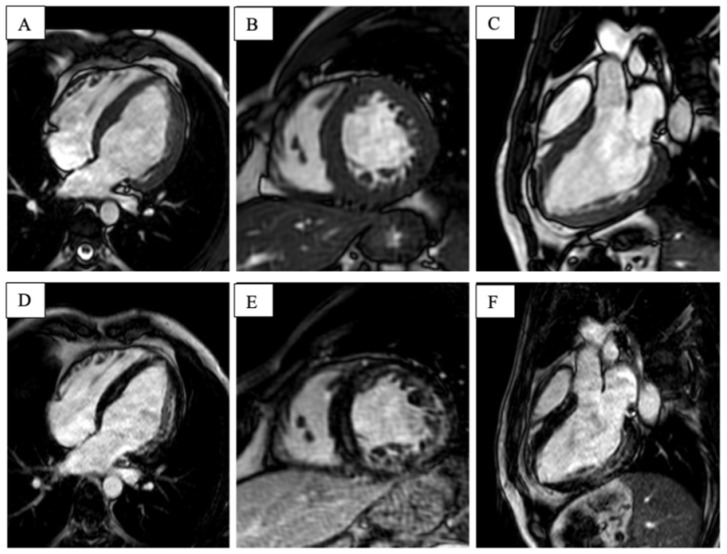
CMR findings in *MTO1*-associated HCM. (**A**) Four-chamber cine view; (**B**) short axis cine view; (**C**) three-chamber cine view; (**D**–**F**) late gadolinium enhancement (LGE) views showing extended LGE with intramyocardial distribution, mainly localized in the antero-lateral wall and in the interventricular septum.

The genetic testing panel identified two heterozygous missense variants in *MTO1* (c.253G>A, p.Gly85Arg; c.1055C>T, p.Thr352Met), and both classified as a variant of unknown significance according to ACMG criteria (Table 1). Follow-up CMR documented extended late gadolinium enhancement with intramyocardial distribution, mainly localized in the anterolateral wall and the interventricular septum (Figure 10).

## 3. Discussion

Inherited NMDs are typically caused by a gene mutation which affects striated muscle and results in progressive muscular weakness, which may cause distinct and sometimes overlapping phenotypes [26]. Cardiac involvement is a major cause of morbidity and mortality among patients with NMDs. Although HCM and DCM may represent the most associated phenotypes in NMDs [1,4,5,17,21,22,27], other cardiomyopathies have been described, including LVNC and restrictive cardiomyopathy (RCM) [21]. 

The need to obtain a specific diagnosis reflects the possibility of performing specific management of both cardiac and non-cardiac involvement [15,28,29]. Thus, identifying a cardiomyopathy phenotype (i.e., hypertrophic, dilated, arrhythmogenic, restrictive) should be followed by identifying the underlying cause [2]. The clinical implication is the possibility to improve and individualize the management of the proband and his family member.

For the diagnostic purpose, the red flags approach has been proposed, which consists of the search for additional diagnostic clues suggesting a specific etiology [12,19,30]. For example, in a patient with DCM, the presence of muscle weakness or increased creatine kinase levels should raise the suspicion of an MD, NMD, or storage disease. Subsequently, the diagnostic work-up should be individualized according to the clinical suspicion. In patients with the suspicion of NMDs, the EMG and MB are often required for the diagnosis. In addition, although MB is considered the mainstay for diagnosing inherited NMDs [20], genetic testing has become of great importance because of its elevated accuracy and less invasive nature [3,21,31]. Furthermore, an unequivocal molecular diagnosis provides information for appropriate genetic counselling, recurrence risk estimate and, eventually, prenatal diagnosis [32].

Our study described the clinical, molecular, and histological characteristics of a consecutive cohort of patients with cardiomyopathy associated with NMDs or MDs. 

First, we described three cases of ACAD9 deficiency. ACAD9 belongs to a family of flavoenzymes associated with the inner mitochondrial membrane and catalyzes the first step of the fatty-acid oxidation cycle. Furthermore, ACADs are required as assembly factors for the mammalian complex I of the mitochondrial respiratory chain [33]. Repp et al. [33] reported that the clinical presentation of ACAD9 patients was dominated by cardiomyopathy, exercise intolerance, and muscle weakness, whereas intellectual disability and neurological symptoms are seldom reported. Patients diagnosed in the first year of life showed a rapid, severely progressive cardiac involvement, and their prognosis was poor compared with patients with a later presentation.

Our study reports two cases of infantile-onset ACAD9 deficiency, showing severe HCM. In particular, *Patient 1* experienced a severe respiratory distress at birth, requiring intensive care therapy and his father was victim of a sudden arrhythmic death. *Patient 1* presented with severe, non-obstructive HCM, but his CMR showed no signs of myocardial fibrosis and the risk for life-threatening arrhythmic events was considered to be low.

We hypothesized that treatment with riboflavin may have changed this patient’s natural history of the disease. Riboflavin has shown promising results among infant-onset patients with ACAD9 deficiency, with a significantly better survival rate than untreated patients [33]. Although its mechanism is unclear, riboflavin could increase flavin adenine dinucleotide (FAD) concentration, favoring ACAD9 folding and showing expanded complex I activity in fibroblast cultures. However, the evidence supporting the role of riboflavin supplementation in ACAD9 patients is inconclusive, and further studies are required to identify the best treatment options. Of interest, *Patient 2* showed a compound heterozygosis, rarely reported in the literature and associated with severe clinical presentation as infantile-onset HCM or mitochondrial multiorgan disorder syndrome (MIMODS) [34]. 

Although MB seldom provides specific findings in patients with a suspected NMD, it can give valuable clues to guide therapeutic management, mainly because the genetic testing can be time-consuming in MD and other NMDs. In our cases of ACAD9 deficiency, the finding of subsarcolemmal aggregates of lipid material, which retained Sudan black, suggested a disorder of fatty acid beta-oxidation. MB can also provide valuable data about the severity of functional impairment, particularly when the interpretation of a rare genetic variant is challenging. In our study, the father of the *Patient 1* was initially believed to be a heterozygous carrier; nevertheless, the severity of the histopathologic findings seemed to conflict with this hypothesis. After a sudden cardiac arrest, muscle biopsy was re-analyzed, and the characterization of muscle cDNA showed the presence of a second *ACAD9* variant, classified as VUS.

Occasionally, different variants in the same gene can be responsible for different cardiomyopathy phenotypes, underlying the need for the etiological diagnosis. For example, *FLNC*, which encodes for filamin C, was originally identified in patients with myofibrillar myopathy presenting with isolated muscle involvement [35]. Subsequently, *FLNC* variants have been found to be associated with different cardiomyopathies (e.g., HCM, RCM) and several skeletal muscle disorders [36].

A similar event occurred for *MYH7*-associated disease. *MYH7* encodes for the beta-myosin heavy chain 7 and this gene is expressed in the myocardium and in skeletal muscles. Variants in *MYH7* were initially described in patients affected with HCM and represent the most common cause of this disorders. However, several other phenotypes have been observed, such as DCM, skeletal myopathy, or mixed phenotypes. Interestingly, we reported two cases of DCM and skeletal myopathy associated with *MYH7* variants. Both patients showed a severe disease phenotype, which lead to heart transplantation in *Patient 4*.

Moreover, we described a patient (*Patient 5*) with a DCM phenotype showing a disease-causing variant in *DES*. Several reports described *DES* variants as associated with different forms of cardiomyopathy and skeletal myopathy [37]. The severity degree of the phenotype varies among patients, ranging from patients with few symptoms to those experiencing severe disease complication or SCD [38]. Here, we described a severe case of DCM with restrictive filling physiology combined with significant muscle involvement who required heart transplantation at an early age. Interestingly, another case carrying the same *DES* variant (p.Arg16Cys) with a RCM phenotype requiring heart transplantation was published [25]. In our case, the diagnosis of desminopathy was strongly suggested by the MB, which revealed the presence of some abnormal desmin aggregates, a typical clue of patients with *DES* variants [38], and then confirmed by genetic testing.

Finally, other two patients *(Patient 6* and *Patient 7)* were diagnosed as having MDs. *Patient 6* was diagnosed with MELAS after he had been implanted with an ICD for SCD risk prevention and *Patient 7* carried a bi-allelic variant in *MTO1*. 

Diagnosis of MD is often complex and requires a high index of suspicion due to the heterogeneous clinical presentation and peculiar inheritance mode [15]. Recently, sudden adult death syndrome has been reported among carriers of *MT-TL1* m.3243A>G variant [39] and according to a systematic review [40] the incidence rate of sudden arrhythmic death among healthy carriers is 2.4 per 1000 person-years. Symptoms, family history of SCD, abnormal ECG or echocardiogram feature, and high mutation load based on blood heteroplasmy were considered high-risk features for sudden arrhythmic death [40]. Recently, a prediction model was validated and found that left ventricular ejection fraction less than 50%, the presence of single, large-scale deletions, and conduction defects were predictors of arrhythmic events [41]. These results highlight the importance of differentiating sarcomeric HCM from other genocopies, in whom tailored risk stratification algorithms may be applied.

## 4. Materials and Methods

### 4.1. Study Design 

The study design included three phases:

1. The enrolment phase occurred between 2010 and 2012 at the Inherited and Rare Cardiovascular Disease Unit of the University of Campania “Luigi Vanvitelli”, Naples, Italy. During the first phase, 425 consecutive patients with cardiomyopathy were identified;
2. Between 2012 and 2013, 24 cardiomyopathy patients showing a persistent increase in serum creatine kinase enzyme were selected for neuromuscular evaluation;
3. Between 2012 and 2022, 11 patients with high suspicion for NMDs underwent a comprehensive neurological, histological, and molecular evaluation, according to the study protocol, and seven patients had a definitive diagnosis of NMD or MD. Preliminary data and the diagnostic work-up is described elsewhere [22].

In the present study, we described the clinical, molecular, and histological characteristics of the seven patients with a definite diagnosis of cardiomyopathies associated with NMDs or MDs.

### 4.2. Study Protocol

Patients were enrolled after informed consent was obtained, according to the procedure established by the Ethics Committee of our institution. All patients underwent a comprehensive cardiovascular and neuromuscular evaluation and genetic testing.

### 4.3. Cardiovascular Protocol

The cardiovascular evaluation included the following components: personal and family history, three-generation pedigree, 12-lead ECG, two-dimensional and Doppler echocardiography, 24-h ECG Holter monitoring, and, when indicated, CMR.

### 4.4. Neuromuscular Protocol

The neuromuscular examination included the evaluation of muscle strength (MS), needle EMG, and MB.

The same physician, a board-certified neurologist, performed all the EMGs. EMGs were performed using a Medtronic Functional Diagnostics EMG apparatus and concentric needle electrodes. Muscles tested by concentric needle differed among patients and were individualized according to the specific clinical question. Insertional activity and spontaneous activity were assessed. Motor unit action potential (MUAP) morphology, duration, and amplitude were compared to our normative data. Interference pattern was characterized as normal or decreased, and recruitment as normal, decreased or early. The time limits of the MUAPs were manually corrected, after inspection of the averaged potentials and re-averaged, thus potentially introducing an element of subjective interpretation. EMG diagnoses were categorized as myopathic, neurogenic, mixed (i.e., neurogenic and myopathic), and normal.

Myopathy was defined according to the EMG criteria as a needle study revealing polyphasic motor units’ potentials of short duration and low amplitude, usually with early recruitment and occasionally with abnormal spontaneous activity [42].

For the MB, target muscles with mild impairment of strength (Medical Research Council grade 4/5) [43] were sampled to avoid end-stage damage. Muscles that showed no abnormality during the examination were less likely to yield meaningful information when biopsied and were also avoided. 

Muscle specimens were obtained by open biopsy under local anesthesia. Muscle tissue was snap-frozen in isopentane pre-cooled in liquid nitrogen at −80 °C, and serial 10 µm thick frozen sections were used for histology and immunohistochemistry. Histochemical stains included haematoxylin/eosin, Gomori trichrome, adenosine triphosphatase (ATPase) at pH 9.6, 4.6 and 4.3, nicotinamide adenine dinucleotide-tetrazolium reductase (NADH-TR), cytochrome oxidase, succinate dehydrogenase (SDH) (COX with SDH), PAS, Sudan III, adenosine monophosphate deaminase (AMPDA), acid phosphatase (AP), and myophosphorylase. IHC was performed to rule out common muscular dystrophies.

The interpretation of muscle biopsies was performed by the same physician with specific expertise in neuromuscular disorders.

### 4.5. Genetic Evaluation

Genetic testing was performed over an extended period from 2010 to 2022. In subjects evaluated between 2010 and 2012, molecular genetic testing was performed using direct sequencing, guided by the clinical phenotype and according to previously described protocols [30]. NGS has been available since 2012. Since that year, and until 2022, all subjects underwent direct Sanger sequencing and extended molecular genetic testing using an NGS panel containing 202 genes (known to be associated with CMPs, channelopathies and nuclear mitochondrial diseases), as previously described [44], and a mitochondrial DNA-targeted NGS panel, followed by whole exome sequencing when first level genetic testing was inconclusive or negative. When indicated, the subjects evaluated before 2012 were re-analyzed using NGS panels and exome sequencing.

## 5. Study Limitations

Recently, new high-resolution respirometry techniques have been proposed to assess oxidative chain function. Nevertheless, these techniques are of limited availability and cannot be performed at our institution. In addition, using large, highly sensitive NGS panels may increase the number of identified variants of unknown significance, whose clinical interpretation could be controversial. Due to the retrospective nature of the case series, we acknowledge that self-reported data may be affected by recall bias and be incomplete or missing.

## 6. Conclusions

We describe the clinical, molecular, and histological characteristics of seven well-characterized patients with NMD and MD presenting with a cardiomyopathy phenotype. Genetic testing, associated with a comprehensive cardiovascular and neuromuscular evaluation, plays a leading role in diagnosing rare NMD and MD, provides information about genetic counselling and clinical expectations, and guides management.

## Figures and Tables

**Table 1:** Genetic variants identified and their pathogenicity classification according to ACMG criteria. Abbreviations: ACMG, American College of Medical Genetics and Genomics; NR, not reported; LP, likely pathogenic; P, pathogenic; VUS, variant of uncertain significance.

Patient	Gene	DNA Position	Protein Position	Frequency GnomAD (Exomes)	ACMG Evaluation	ACMG Criteria
***Patient 1***	*ACAD9*	c.1240C>T	p.Arg414Cys	0.0000119	P	PP5, PM5, PP3, PM1, PM2, PP1
	*ACAD9*	c.1240C>T	p.Arg414Cys	0.0000119	P	PP5, PM5, PP3, PM1, PM2, PP1
***Patient 2***	*ACAD9*	c.1240C>T	p.Arg414Cys	0.0000119	P	PP5, PM5, PP3, PM1, PM2, PP1
	*ACAD9*	c.1646G>A	p.Arg549Gln	0.00000802	VUS	PP3, PM2, BP1
***Patient 3***	*MYH7*	c.1325G>A	p.Arg442His	0.0000119	P	PS3, PM1, PM5, PP3, PP5, PM2, PP1
	*MYH7*	c.3866G>A	p.Arg1289Gln	0.00000795	VUS	PP3, PM2, PP1, PP2
***Patient 4***	*MYH7*	c.1357C>T	p.Arg453Cys	NR	P	PP5, PM5, PP3, PM1, PM2
***Patient 5***	*DES*	c.46C>T	p.Arg16Cys	NR	LP	PP3, PM1, PP5, PM2
***Patient 6***	*MT-TL1*	m.3243A>G	N/A	NR	P	PM2, PP3, PS4, PP5, PM5
***Patient 7***	*MTO1*	c.253G>A	p.Gly85Arg	0.00000795	VUS	PP3, PM2, BP1
	*MTO1*	c.1055C>T	p.Thr352Met	0.00000398	VUS	PP3, PM2, BP1

## Data Availability

The data is contained within the article. Additional information is available on request from the corresponding author.
